# Five successive waves of SARS-CoV-2 infection in the Central African Republic: a prospective observational study from 2020-2022

**DOI:** 10.11604/pamj.2023.46.120.39511

**Published:** 2023-12-28

**Authors:** Clotaire Donatien Rafaï, Luc Salva Heredeibona, Ernest Lango-Yaya, Roseline Darnicka Belizaire, Oscar Senzongo, Placide Mbala, Maurel Ouoko Fa-Ti-Gbia, Javan Allon Bengba, Simon Pounguinza, Jephté Estimé Kaleb Kandou, Daniel Yvon Gonessa, Wilfried Koyaweda, Ulrich Vickos, Ginette Claude Kalla, Wilfried Sylvain Nambei, Pierre Somse, Laurent Bélec, Gérard Grésenguet, Boniface Koffi, François-Xavier Mbopi-Keou

**Affiliations:** 1 Laboratoire National de Biologie Clinique et de Santé Publique, Bangui, République Centrafricaine,; 2 Faculté des Sciences de la Santé, Université de Bangui, Centrafrique de la Santé et de la Population, Bangui, République Centrafricaine,; 3 Ministry of Health and population, Bangui, Central African Republic,; 4 World Health Organization, Bangui, Central African Republic,; 5 National Institute of Biomedical Research of Kinshasa, Kinshasa, Democratic Republic of the Congo,; 6 Laboratoire de Virologie, Hôpital Européen Georges Pompidou, Université Paris Cité, Paris, France,; 7 Faculty of Medicine and Biomedical Sciences, University of Yaoundé I, Yaoundé, Cameroon

**Keywords:** SARS-CoV-2, COVID-19, Central African Republic

## Abstract

**Introduction:**

the National Laboratory of Clinical Biology and Public Health (NLBPH) in Bangui in the Central African Republic (CAR) carries out the vast majority of molecular screening tests for SARS-CoV-2 infection nationwide. This study aimed to show the contribution of molecular diagnosis and genomic surveillance in monitoring the evolution of longitudinal variations of the SARS-CoV-2 infection epidemic in CAR between 2020 and the end of 2022.

**Methods:**

this is an observational study on the variations in the prevalence of detection of SARS-CoV-2 by RT-PCR at the NLCBPH from nasopharyngeal samples taken prospectively over a period of 3 years since the beginning of the COVID-19 epidemic. A subgroup of SARS-CoV-2 positive samples was selected for molecular sequencing performed by Illumina® and MinIon® at the National Institute for Biomedical Research in Kinshasa, Democratic Republic of the Congo.

**Results:**

from March 2020 to December 31^th^, 2022, 88,442 RT-PCR tests were carried out (4/5 of the country) and detected 9,156 cases of SARS-CoV-2 infection in 5 successive waves. The average age of the patients was 39.8 years (extremes ranging from to 92 years). Age(P=0.001), sex(P=0.001) and symptom presentation(P=0.001) were significantly associated with RT-PCR test positivity. Among the different variants identified during successive waves, the Omicron variant predominated during the last two waves.

**Conclusion:**

this prospective study over a period of 3 years, marked by 5 successive waves, made it possible to report that age, sex and the presence of clinical symptoms are associated with RT-PCR positivity. Among the different variants identified during successive waves, the Omicron variant predominated during the last two waves.

## Introduction

Leaving Wuhan at the end of November 2019, and declared a pandemic on March 11^th^, 2020, COVID-19 has been one of the most devastating pandemics of the last two centuries. But with the use of effective vaccines since the end of 2020 and the low tropism of the Omicron variant for the lower airways [1], its impact is increasingly limited even if the latest outbreaks reported in China are a reminder of vigilance [2,3]. The African continent has not been spared, although with the exception of countries like South Africa which have large capacities for RT-PCR testing, fewer cases and deaths have been reported compared to the rest of the world [4,5]. The Central African region has been able to demonstrate resilience while facing the pandemic [5]. With the support of partners and Africa CDC, many countries acquired diagnostic capabilities by RT-PCR SARS-CoV-2 [6]. The Central African Republic diagnosed its first case on March 14^th^, 2020 and to date 15,363 cases have been diagnosed, including 113 deaths out of 114,736 RT-PCR tests carried out over the past three years marked by 5 waves with 4 predominant variants. The country has also recorded so far 2,444,885 people vaccinated [7-9]. The National Laboratory of Clinical Biology and Public Health (NLCBPH) is one of the main reference laboratory of the country and has diagnosed 4/5 of the RT-PCR tests in the Country, in addition to hosting almost all of the databases of the country. The aim of this study is to report epidemiological and virological data for the diagnosis of COVID-19 carried out by the NLCBPH over the past three years.

## Methods

**Study setting**: the NLCBPH, one of the two Main Reference Laboratories of the Country, and which is attached to the Ministry of Health and Population of the Central African Republic served as the setting for this study. This is a descriptive analytical study of laboratory databases collected using national COVID-19 surveillance sheets.

**Recruitment and analysis of samples**: the study was carried out from March 2020 to December 31, 2022. The RT-PCR diagnostic samples were extracted manually from April 2020 to July 2022 using the QIAGEN kit (QIAGEN GmbH Extraction KIT, Hilden, Germany). The automated method was performed by the MagMax Kit on King Fisher from THERMOSCIENTIF FISHER in addition to the manual method, from May 2022. For RT-PCR, the Da An Gene kit was used (Da An Gene Co., Ltd. Of Sun Yat-sen University, Guangzhou, Guangdong, P. R. China) following the manufacturers' instructions [7]. Samples tested and confirmed positive with CT values below 30 and which presented details of interest (during waves, associated with severe cases or in vaccinated persons) were referred for sequencing by Illumina or MinIon at the National Institute of Biomedical Research in Kinshasa of Kinshasa.

**Data management and analysis**: data were analyzed using Epiinfo version 3.3.2 software and the extracted databases were processed in Excel and Word as needed. Categorical variables were presented as frequencies and percentages. Continuous variables were presented as means (and standard deviation). level of statistical significance was set at a p-value of <0.05.

**Identification of pandemic waves**: operationally, we speak of a pandemic wave when we observe a rapid, exponential increase in the number of contaminations over a sustained period. On a technical level, the positivity of RT-PCR evolves until it reaches a peak before gradually decreasing. Before the introduction of vaccines, serious cases requiring hospitalization increased to the point of causing capacity overruns. With the intensification of vaccination campaigns, new waves result from the introduction of new variants or sub-variants presenting a new antigenic particularity. In our study, we identified the waves by the peaks of RT-PCR positivity which characterized the three years of health monitoring.

**Ethical and administrative procedures**: this study which was part of the national response against COVID-19 was approved by the Institutional Ethical Review Committee of the Ministry of Health and Population of the Central African Republic (CAR). Administrative authorization was obtained from the Minister of Health and Population of the CAR. Voluntary informed consent was obtained from the patients. In addition, we received ethical clearance from the Ethics and Scientific Committee of the Faculty of Health Sciences (N32/FACSS/CES.2020).

## Results

**Description of the study population**: the average age of patients was 39.83 years with a minimum of 1 and a maximum of 92 years old. The most representative age group were those of 31 to 40 years old (n=25175, 28.47%), followed by those of 40 to 50 years old (n=22340, 25.26%). The male gender was the most represented (n=61252) with a percentage of 69.27% giving a sex ratio of 2.61 (Table 1).

**Table 1:** demographic and epidemiological characteristics of the study population

	Results				Total	p-value 0,05
Settings	Positive	Percentage %	Negative	Percentage (%)		
**Average age (year) ± SD**	38,68 ±14,24		40,00 ± 13,84		39,86 ±13,90	
**Median age (year)**	38,00		40,00		40,00	
**Age in category (year)**						0,001
0 E11 month	2	0,96	206	99,04	208	
1 E10 years	262	13,60	1665	86,40	1927	
11 E20 years	569	12,04	4158	87,96	4727	
21 E30 years	1589	10,96	12915	89,04	14504	
31 E40 years	2963	11,77	22212	88,23	25175	
40 E50 years	2005	8,86	20335	91,14	22340	
51 years and over	1766	9,04	17775	90,96	19541	
TOTAL	9156	10,35	79266	89,65	88422	
**Gender**						
Male	6133	10,01	55119	89,99	61252	0,001
Female	3023	11,13	24147	88,87	27170	
Sex Ratio H/F=2,61						
TOTAL	9156	10,35	79266	89,65	88422	
**Patient status**						0,001
Symptomatic	1406	16,79	6966	83,21	8372	
Asymptomatic	7750	9,68	72300	90,32	80050	
TOTAL	9156	10,35	79266	89,65	88422	
**Year**						
**2020**						0,137
Male	2099	19,89	8454	80,11	10553	
Female	771	19,07	3272	80,93	4043	
Sex Ratio H/F=2,61	2870	19,66	11726	80,34	14596	
**Year**						
**2021**						0,001
Male	2580	11,16	20547	88,84	23127	
Female	1574	14,09	9500	85,91	11174	
Sex Ratio H/F=2,61	4154	12,11	30147	87,89	34311	
**Year**						
**2022**						0,056
Male	1454	5,27	26118	94,73	27572	
Female	678	5,67	11275	94,33	11953	
Sex Ratio H/F=2,61	2132	5,39	37393	94,51	39525	

**Figure 1:**
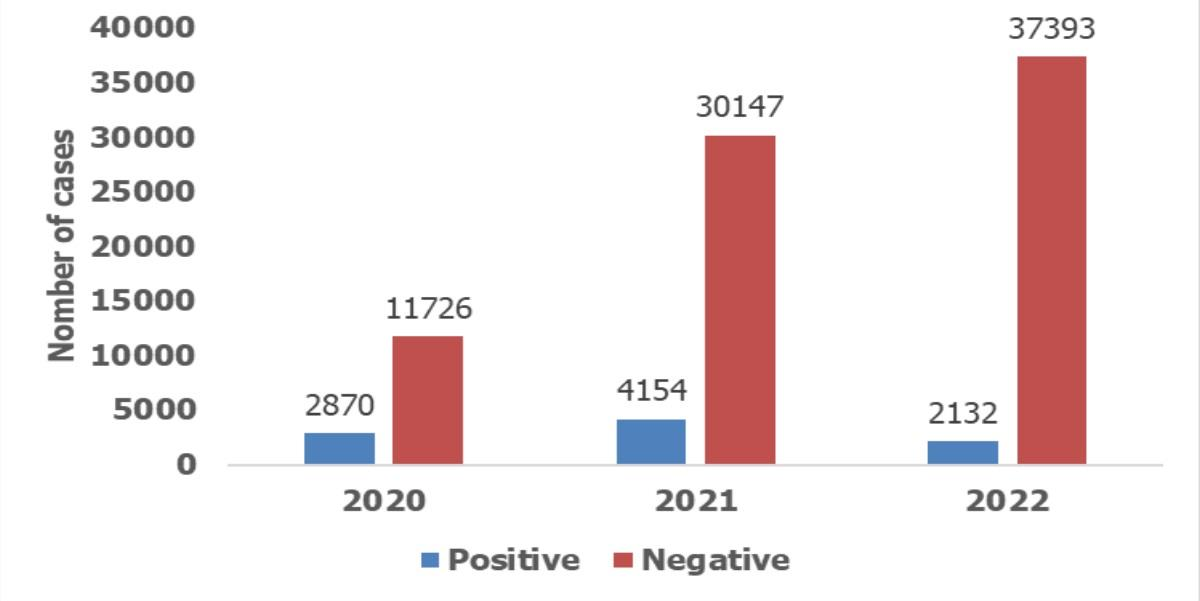
data from RT-PCR tests carried out over the last 3 years by the National Laboratory for Clinical Biology and Public Health

**Analysis of samples**: from March 2020 to December 31, 2022, SARS-CoV-2 RT-PCR tests were carried out and made it possible to detect 9,156 cases of Covid-19. Age, sex and presentation of symptoms were significantly associated with positive RT-PCR tests (p=0.001) (Figure 1).

**Figure 2:**
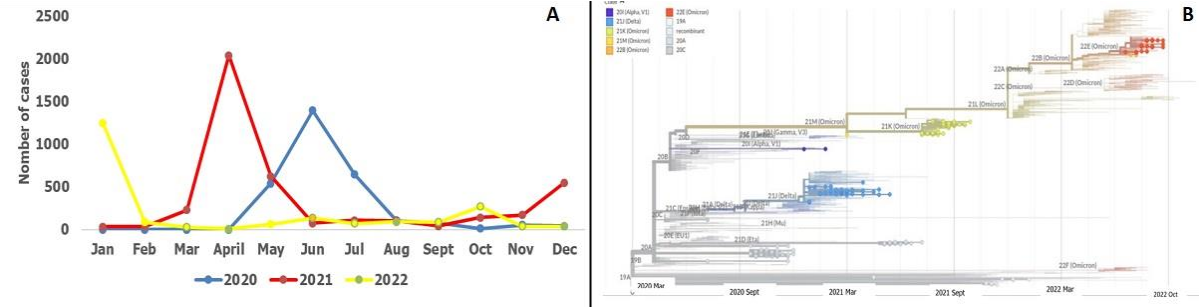
A,B) evolution of SARS-CoV-2 infection in the Central African Republic over the past 3 years

**Figure 3:**
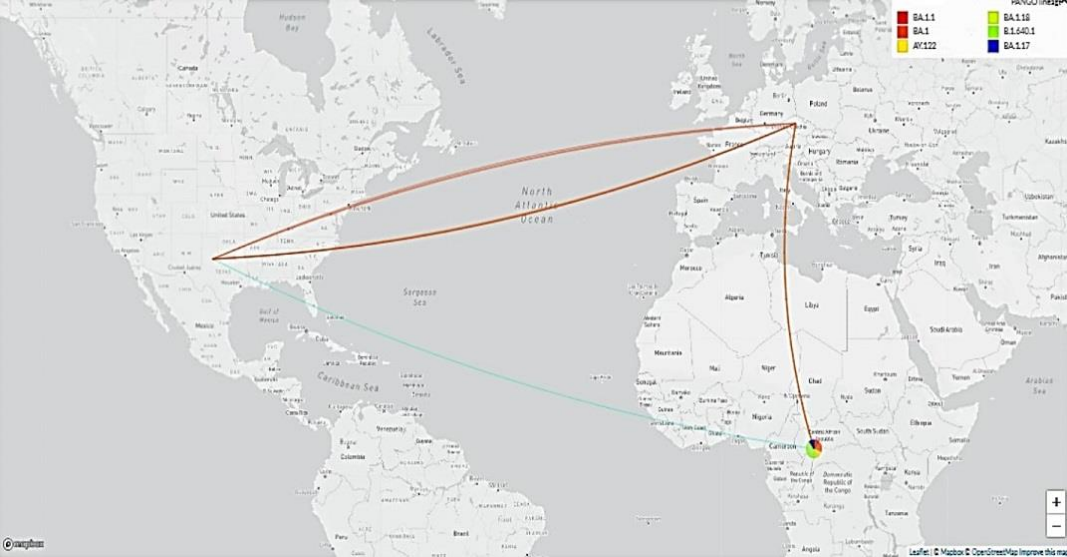
origin of SARS-CoV-2 variants circulating between March 2020 and December 2022

**Evolution of SARS-CoV-2 infection in the Central African Republic**: as shown in Figure 2, from 2020 to 2022, the CAR has experienced 5 successive waves, the peaks of which were reached in June 2020, April 2021, December 2021, June 2021 and October 2022. Out of 202 samples sent for sequencing, 197 sequences were obtained and made it possible to detect 10 variants, the most predominant of which being Alpha, Delta and Omicron. The latter predominated during the 4th wave and was the only variant found during the 5^th^ wave (Figure 2 and Figure 3).

## Discussion

The average age reported in our study is 40, which reflects the reality of the African context where the population is predominantly young [7,10]. Age, sex and onset of symptoms were associated with positive SARS-CoV-2 RT-PCR tests. These risk factors are known in both European and African series. Lango Yaya and Manirakiza in 2020 and 2021 reported strong positivity among men and age groups ranging from under 50 years old. This is an active population that was very exposed in this context of partial confinement, since they were working without correct observance of barrier measures.

Of the 88,422 SARS-CoV-2 RT-PCR tests performed from 2020 to 2022, 9,156 were positive, representing a positivity rate of 10.35%. Higher prevalences have been reported by Lango Yaya and Manirakiza in the Central African Republic between 2020 and 2022 [7,10]. This corroborates well with our 2020 data which reports a high RT-PCR positivity rate (24.4%). It should be noted that in CAR at the early stage of the pandemic, RT-PCR tests were rare commodities reserved for symptomatic cases and mass screenings were carried out by serological and antigenic tests, the positives of which were confirmed by RT. -PCR [11-16]. Hence this strong positivity of the RT-PCR tests. Furthermore, with vaccination and the implementation of international health regulations including systematic RT-PCR SARS-CoV-2 tests performed on travellers, the positivity rate dropped down over the months due to the effect of mass screening of mostly asymptomatic people [5].

Central African Republic has experienced 5 successive waves like most countries in sub-Saharan Africa, but the 4th wave was underdiagnosed due to pandemic fatigue which followed the third wave. This pandemic appears as a reminder of how the impact of the COVID-19 pandemic can be felt in low-resource countries whose resilience capacities can be gradually blunted as this pandemic continues. This therefore justifies the lifting of certain measures (such as the requirement of RT-PCR tests for travellers) rendered useless by the improvement in vaccination coverage but which were still in force and contributed to the waste of tests. This is all the more true since travelers who are mostly vaccinated should not be considered either as people at risk or as priority targets.

The variants detected corresponded to those identified during upsurges in the world and especially in the sub-region almost at the same time. The CAR is one of the countries with the largest international community of United Nations agents accustomed to frequent movements because of their status. This increases the risk of importation of COVID-19 variants. We believe that vaccination coverage and the implementation of genomic surveillance will minimize the risk of a large-scale spread of the virus.

## Conclusion

This study enabled to understand the history of the COVID-19 pandemic in the Central African Republic. The Central African Republic, although slightly impacted by the pandemic, experienced high positivity rates during the 5 waves. The Omicron variant, which predominated during the last two waves, had little impact on the health system due to the improvement in vaccination coverage.

### What is known about this topic

- *COVID-19 has been one of the most devastating pandemics of the last two centuries;*
- *The use of effective vaccines since the end of 2020 and the low tropism of the Omicron variant for the lower airways have limited its impact;*
- *The African continent has not been spared, although with the exception of countries like South Africa which have large capacities for RT-PCR testing, fewer cases and deaths have been reported compared to the rest of the world*.

### What this study adds

- *This study enabled to better understand the history of circulation of SARS-CoV-2 in the Central African Republic;*
- *Age, sex and symptom presentation were significantly associated with RT-PCR test positivity;*
- *The predominant variants detected were Alpha, Delta and Omicron*.
